# Loss of dental pulp potentially increased the risk of Alzheimer's dementia

**DOI:** 10.1016/j.jds.2024.07.006

**Published:** 2024-07-16

**Authors:** Seung Hyun Son, Sang-woo Lee, Gehoon Chung

**Affiliations:** a Department of Oral Physiology, School of Dentistry, Seoul National University, Seoul, Republic of Korea; b Dental Research Institute, Seoul National University, Seoul, Republic of Korea

**Keywords:** Alzheimer's dementia, Systemic disease, Tooth extraction, Pulp, Pulpectomy, Data mining

## Abstract

**Background/purpose:**

Chronic periodontitis and tooth loss contribute to cognitive decline. Since many biological processes are shared by loss of teeth and loss of pulps, this study investigated the potential association between loss of pulp and the development of dementia.

**Materials and methods:**

A retrospective cohort analysis was conducted to investigate the association between dental treatment and the development of dementia. The records of dental treatment during the 10 years prior to the first diagnosis of dementia were extracted from the Elderly Cohort Database of the National Health Information Sharing Service of Korea. The independence of dementia compared to the number of pulps or teeth removed was evaluated using the chi-squared test. The subjects were grouped by the number of teeth or pulps treated, and their odds ratio for dementia was calculated.

**Results:**

Analysis of 591,592 sessions for pulpectomy and 710,722 sessions for tooth extraction from 558,147 individuals revealed a significant association with Alzheimer's dementia, but not with vascular or unspecified dementia. The number of dementia patients based on the number of pulps or teeth extracted were significantly different across age groups. The odds ratios demonstrated a tendency to increase with the number of dental treatments and decrease with age at the time of diagnosis of dementia. The number of pulps removed to achieve a notable impact on Alzheimer's dementia was found to be lower than the number of teeth extracted.

**Conclusion:**

The loss of pulp increased incidence of Alzheimer's dementia, with the impact being more pronounced in younger geriatric groups.

## Introduction

Dementia is progressive and debilitating neurodegenerative disorders, with Alzheimer's disease accounting for approximately 60–70% of cases. The common characteristics include cognitive decline, memory impairment, and changes in behavior and personality, leading to significant functional impairment and a diminished quality of life. Because definitive treatment has not been developed, risk assessment and prevention are crucial. Risk factors associated with the development of dementia includes age, genetics, lifestyle choices, cardiovascular health, education level, and social engagement.

A number of studies have demonstrated a correlation between poor oral health and an increased risk of cognitive decline and dementia.^1^, ^2^, ^3^, ^4^, ^5^, ^6^, ^7^, ^8^, ^9^ However, the evidence supporting the role of oral health in the development of dementia has been mixed. Some studies have suggested that dementia may develop as a consequence of periodontal problems resulting from poor oral hygiene.^10^, ^11^, ^12^, ^13^, ^14^, ^15^, ^16^ Chronic inflammation and infection in the oral cavity may trigger neuroinflammation and oxidative stress, contributing to neurodegenerative processes and cognitive impairment. Interestingly, a population cohort study reported that dementia was inversely correlated to periodontitis and more often observed in those with removable dentures, suggesting that loss of teeth and edentulousness *per se* rather than inflammation might be the contributing factor to the development of dementia.^1^, ^2^, ^3^, ^4^, ^5^^,^^17^^,^^18^ A potential confounding factor is reduced chewing ability,^19^ as subsequent malnutrition may have contributed to the development of dementia, as was the case with swallowing dysfunction.^20^ However, oral rehabilitation did not demonstrate a clear improvement in cognitive function,^21^ and the mechanism linking masticatory function to cognitive function remains unclear.

While previous research has focused on the impact of tooth loss, the current study investigated the correlation between pulpal loss and dementia. Although the clinical approaches for managing pulp loss and tooth loss may differ, the underlying biological processes share many similarities. Both conditions are often the result of chronic inflammatory processes, and the soft tissue removed by pulp removal and tooth extraction is the same. Therefore, the loss of pulp may contribute to the development of dementia in a manner similar to the loss of teeth. This hypothesis was tested by analyzing data from the Elderly Cohort Database (ECD) of the National Health Information Sharing Service, provided by the National Health Insurance Service of Korea. In particular, the number of dementia patients was compared in relation to the number of pulp-removed or teeth extracted within 10 years prior to the onset of Alzheimer's, vascular or unspecified dementia.

## Materials and methods

### Data source and ethical considerations

The Elderly Cohort Database (ECD) of the National Health Information Sharing Service (NHISS) is a retrospective cohort of individuals over 60 years of age at the end of 2002. These individuals were randomly selected from the entire Korean population registered in the National Health Insurance Service. The cohort spans 18 years, from 2002 to 2019. Those diagnosed with dementia before 2012 or those who died before the end of the cohort were excluded from the study.

This study adhered to the guidelines outlined in the Strengthening the Reporting of Observational Studies in Epidemiology (STROBE) statement. All procedures were reviewed and approved by the Institutional Review Board (S-D20210026). The NHISS data was thoroughly anonymized in accordance with the guidelines of the Personal Information Protection Act of Korea.

### Study design and selection of the subjects

The records of dental sessions for pulpectomy were extracted from the treatment table of ECD using the code U0101. The individuals who were diagnosed with Alzheimer's dementia, identified with the Korean standard classification of diseases (KCD) code F00 or G30, for the first time in 2012, were selected from the pulp removal session data. A same number of healthy subjects, who had not been diagnosed with Alzheimer's dementia throughout the entire cohort period, were also selected randomly from the pulp removal data set, matching the propensity score based on age and sex^22^ using an R package, MatchIt.^23^ The number of pulps removed was counted by the number of FDI notations on the treatment records. The counting was valid only when an individual had dental sessions for pulpectomy within a 10-year period prior to 2012, between 2002 and 2011. The aforementioned selection processes were repeated for the years 2013 through 2019. Subsequently, all data from 2012 to 2019 were merged to ascertain the number of pulps removed during the 10 years prior to the initial diagnosis of Alzheimer's dementia, with an equal number of healthy controls being included in each year.

The procedure was repeated with KCD code F01 to select individuals for the vascular dementia group and KCD code F03, F028, G310, and G318 for the unspecified dementia group. Thereafter, the equal number of subjects were selected, and the number of pulps removed within 10 years prior to diagnosis was counted in each group. Finally, the entire process was repeated, with the number of extracted teeth instead of pulps removed. This was done using treatment code U441 for those diagnosed with Alzheimer's, vascular, and unspecified dementia, respectively.

### Statistical analysis

All analyses were conducted using the R software (version 4.4.0; R Foundation, Vienna, Austria). A chi-squared test was performed to investigate whether the development of dementia is independent of the dental treatment history. The subjects were classified into five groups based on the number of pulps removed or teeth extracted during the 10-year period prior to the first diagnosis of dementia: 0, 1–5, 6–10, 11–15, and 16 or more. To ascertain whether this relationship varies across different age groups, the subjects were further categorized into seven age groups: less than 60 years, 60–64 years, 65–69 years, 70–74 years, 75–79 years, 80–84 years, and 85 years or older. Odds ratios associating dental treatment with dementia were calculated separately for each age group.

## Results

### Demographic characteristics of study population

Among the 558,147 individuals included in the ECD, 97,942 were diagnosed with Alzheimer's dementia, 21,117 were diagnosed with vascular dementia, and 43,891 were diagnosed with unspecified dementia. Following the exclusion of individuals who had died prior to the conclusion of the cohort period, the remaining subjects were classified as follows: 63,547 Alzheimer's dementia cases, 12,469 vascular dementia cases, and 27,227 cases of unspecified dementia.

**Figure 1:**
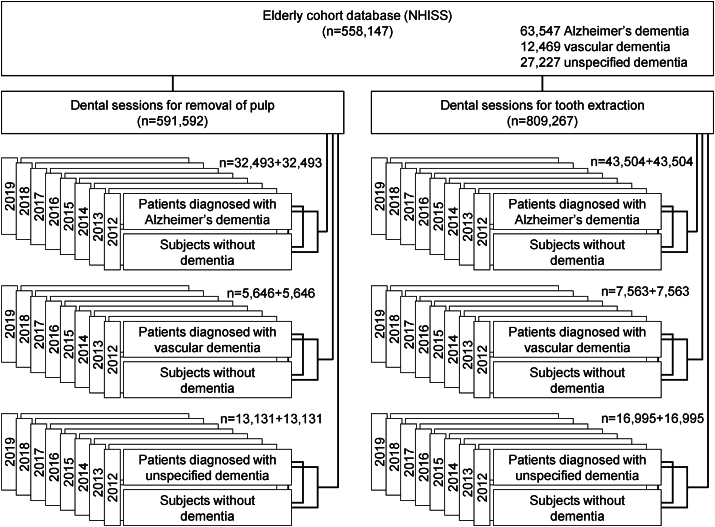
Selection of the study population from the elderly cohort database of the National Health Information Sharing Service (NHISS) of the Republic of Korea.

Total of 32,493 dementia patients and 32,493 controls without dementia were extracted from investigation of 591,592 visits for pulpectomy. Similarly, 5646 and 13,131 subjects with vascular or unspecified dementia, respectively, were selected with equal number of non-dementia subjects each. Investigation of 710,722 visits for tooth extraction revealed 43,504 patients with Alzheimer's dementia, 7563 with vascular dementia, and 16,995 with unspecified dementia, with respective age-and-sex matched controls (Fig. 1).

### Chi-squared test for independence

**Figure 2:**
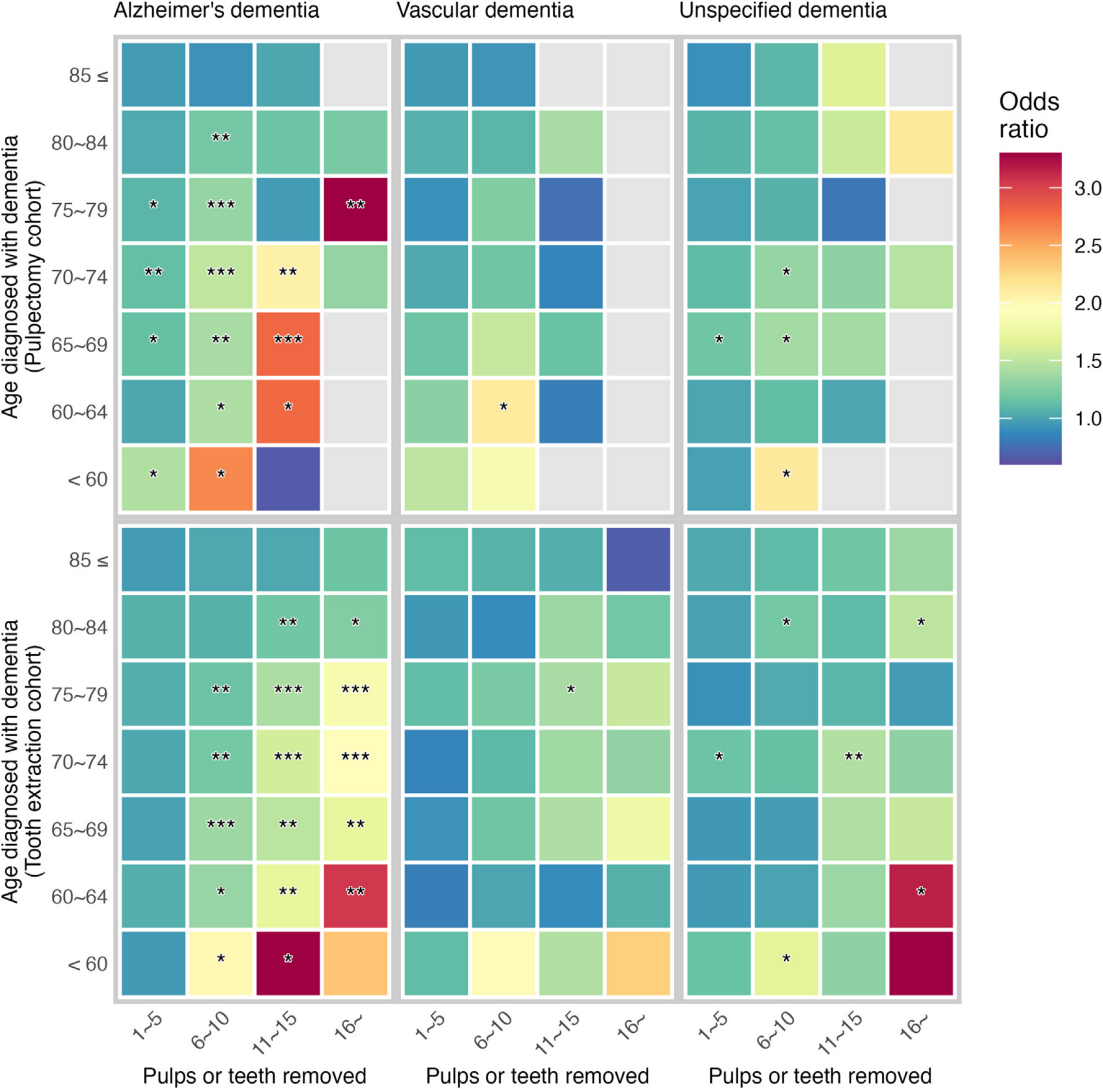
Heatmap showing odds ratios (OR) of pulp removal (upper panel) or tooth extraction (lower panel) for dementia within each age groups. The rectangles were colored by OR indicated in the color scales, or greyed when OR was not available. Asterisks were added where OR was statistically significant (∗*P* < 0.05; ∗∗*P* < 0.01; ∗∗∗*P* < 0.001).

The number of Alzheimer's dementia patients and healthy control subjects varied significantly by the number of pulp removals (χ^2^ = 63.896, *P* < 0.001). When the subjects were further categorized into seven age groups, significant differences were observed across age groups under 79 years old. Tooth extraction data also revealed significant different numbers of subject with or without Alzheimer's dementia (χ^2^ = 146.191, *P* < 0.001). Significant differences were observed across all age groups, with the exception of those aged 85 years and older (Table 1). Interestingly, the increase in Alzheimer's dementia was observed when only a few pulps were removed, whereas the impact of tooth extraction was visible when more than six teeth were extracted (Fig. 2).

**Table 1:** Chi-squared test for independence between dementia and non-dementia groups.

Age group	Pulp removed							Tooth extracted						
	0	1∼5	6∼10	11∼15	16∼	ꭓ^2^	*P*	0	1∼5	6∼10	11∼15	16∼	ꭓ^2^	*P*
Alzheimer's														
All	7662/8149	22,363/22,335	2205/1816	233/175	30/18	63.896	0.000	7307/7759	24,974/25,775	7870/7372	2467/1995	886/603	146.191	0.000
85 ≤	1266/1218	2512/2509	198/206	18/17	1/0	1.861	0.761	1125/1103	3343/3399	1249/1199	364/349	123/103	3.579	0.466
80∼84	2052/2129	5558/5591	524/451	46/41	7/6	7.287	0.121	1850/1975	6564/6575	2288/2294	714/643	244/207	10.803	0.029
75∼79	2064/2232	6375/6345	644/525	55/62	14/2	28.162	0.000	1878/2049	6834/7087	2205/2069	730/562	251/148	64.794	0.000
70∼74	1223/1379	4281/4271	476/356	55/30	7/6	34.102	0.000	1292/1386	4408/4635	1265/1133	376/252	143/78	59.865	0.000
65∼69	679/768	2254/2232	224/181	42/17	1/2	21.074	0.000	718/762	2311/2471	550/431	185/132	83/51	37.600	0.000
60∼64	275/287	1081/1110	119/87	16/6	0/1	11.156	0.025	301/334	1198/1261	269/223	81/52	33/12	23.753	0.000
<60	103/136	302/277	20/10	1/2	0/1	10.303	0.036	143/150	316/347	44/23	17/5	9/4	16.667	0.002
Vascular														
All	1296/1350	3896/3912	419/348	29/33	6/3	8.965	0.062	1306/1328	4376/4548	1285/1220	442/345	154/122	20.851	0.000
85 ≤	165/159	327/330	29/30	0/2	0/0	.	.	137/147	454/436	150/150	52/53	12/19	2.306	0.680
80∼84	311/326	852/842	83/80	8/6	1/0	1.753	0.781	316/305	1001/1026	325/354	125/89	37/30	8.529	0.074
75∼79	329/314	1041/1088	131/100	8/10	2/0	7.770	0.100	314/356	1151/1169	363/339	121/98	49/36	7.997	0.092
70∼74	233/240	821/822	88/77	5/6	0/2	2.928	0.570	242/227	843/925	233/199	79/54	28/20	12.991	0.011
65∼69	144/166	493/487	52/39	6/6	3/0	6.455	0.168	163/162	509/550	121/102	43/30	18/10	7.810	0.099
60∼64	74/92	265/257	29/17	2/3	0/1	6.405	0.171	88/78	295/315	74/65	17/17	6/5	1.932	0.748
<60	40/53	97/86	7/5	0/0	0/0	.	.	46/53	123/127	19/11	5/4	4/2	3.470	0.482
Unspecified														
All	3050/3188	9089/9070	891/783	91/81	10/9	10.675	0.030	2960/3059	10,002/10,283	2882/2694	851/728	300/231	30.407	0.000
85 ≤	384/363	767/798	60/52	7/4	0/0	.	.	340/357	988/1010	365/346	105/93	40/31	3.032	0.552
80∼84	624/664	1798/1776	172/161	16/11	2/1	3.000	0.558	587/612	2047/2156	742/645	210/198	79/55	14.783	0.005
75∼79	763/763	2291/2299	252/236	19/24	2/5	2.406	0.662	723/693	2475/2592	807/748	244/216	68/68	7.280	0.122
70∼74	603/663	1944/1925	209/173	26/22	4/3	6.806	0.147	554/634	2047/2005	517/518	160/127	50/44	10.001	0.040
65∼69	356/410	1242/1212	114/95	18/15	2/0	8.174	0.085	410/401	1307/1355	251/253	82/56	41/26	9.230	0.056
60∼64	211/214	786/789	61/55	5/5	0/0	.	.	218/215	851/878	156/154	41/30	16/5	7.921	0.094
<60	109/111	261/271	23/11	0/0	0/0	.	.	128/147	287/287	44/30	9/8	6/2	6.020	0.198

Data are presented as number of dementia patients/number of non-dementia individuals unless noted otherwise. ꭓ^2^: chi-squared value.

It is noteworthy that the number of vascular dementia patients and healthy control subjects was independent of the number of pulp removals (χ^2^ = 8.965, *P* = 0.062). This independence was observed across all age groups. In contrast, the results from the tooth extraction data revealed a significant difference in the number of vascular dementia patients and healthy controls (χ^2^ = 20.851, *P* < 0.001). However, independence was observed only in the age group 70–74 (χ^2^ = 12.991, *P* = 0.011) when further categorized (Table 1).

The number of unspecified dementia patients and healthy control subjects were independent of the number of pulp removals (χ^2^ = 10.675, *P* = 0.030). However, this difference was not significant when the analysis was conducted within separate age groups. The data on tooth extractions revealed a significant difference in the number of unspecified dementia patients and healthy controls (χ^2^ = 30.407, *P* < 0.001). It is noteworthy that the age groups 70–74 (χ^2^ = 10.001, *P* = 0.040) and 80–84 (χ^2^ = 14.783, *P* = 0.005) demonstrated significant differences when further categorized.

### Differential impact of pulp removal across age groups

The odds ratio (OR) for pulp removal in relation to the prevalence of Alzheimer's dementia exhibited variability based on the number of pulps removed and the age of the subjects. Among individuals aged 60 to 84, those who had 6 to 10 pulps removed within the 10 years prior to diagnosis exhibited 1.205 to 1.508 times higher odds of developing Alzheimer's disease. The removal of 11–15 pulps yielded ORs between 2.067 and 2.794 for individuals aged 60 to 74 but did not induce a significant increase in other age groups. The removal of one to five pulps demonstrated a slight increase in the OR (1.087–1.142, *P* < 0.05) only in the age groups 65 to 79. It is noteworthy that the ORs for Alzheimer's disease in the 1–5 and 6–10 pulp removal groups were significantly higher in individuals under 60 compared to other age groups (1.440 and 2.641, *P* < 0.05). The removal of 16 or more pulps demonstrated a statistically significant association only in the age group 75–79 (OR: 7.570, 95% CI: 1.718–33.347, *P* = 0.002) (Fig. 2 and Table 2).

**Table 2:** Odds ratios (OR) of pulp removal for dementia.

	Pulp removed	Alzheimer's						Vascular						Unspecified					
		Dmtia	Ctrl	OR	95% CI	*P*	Sig.	Dmtia	Ctrl	OR	95% CI	*P*	Sig.	Dmtia	Ctrl	OR	95% CI	*P*	Sig.
85 ≤	1∼5	2512	2509	0.963	[0.875, 1.060]	0.445	n.s.	327	330	0.955	[0.732, 1.246]	0.734	n.s.	767	798	0.909	[0.763, 1.082]	0.281	n.s.
	6∼10	198	206	0.925	[0.749, 1.141]	0.466	n.s.	29	30	0.932	[0.535, 1.623]	0.802	n.s.	60	52	1.091	[0.733, 1.624]	0.669	n.s.
	11∼15	18	17	1.019	[0.523, 1.986]	0.957	n.s.	0	2	0.000	.	0.151	n.s.	7	4	1.654	[0.480, 5.698]	0.420	n.s.
	16∼	1	0	.	.	0.327	n.s.	0	0	.	.	.	·	0	0	.	.	.	·
80∼84	1∼5	5558	5591	1.031	[0.961, 1.107]	0.394	n.s.	852	842	1.061	[0.884, 1.273]	0.526	n.s.	1798	1776	1.077	[0.948, 1.224]	0.252	n.s.
	6∼10	524	451	1.205	[1.048, 1.386]	0.009	∗∗	83	80	1.088	[0.771, 1.534]	0.633	n.s.	172	161	1.137	[0.893, 1.447]	0.297	n.s.
	11∼15	46	41	1.164	[0.761, 1.781]	0.484	n.s.	8	6	1.398	[0.479, 4.074]	0.538	n.s.	16	11	1.548	[0.713, 3.361]	0.266	n.s.
	16∼	7	6	1.210	[0.406, 3.608]	0.731	n.s.	1	0	.	.	0.306	n.s.	2	1	2.128	[0.192, 23.529]	0.528	n.s.
75∼79	1∼5	6375	6345	1.087	[1.014, 1.164]	0.019	∗	1041	1088	0.913	[0.765, 1.089]	0.313	n.s.	2291	2299	0.997	[0.888, 1.119]	0.953	n.s.
	6∼10	644	525	1.327	[1.165, 1.510]	0.000	∗∗∗	131	100	1.250	[0.924, 1.692]	0.148	n.s.	252	236	1.068	[0.871, 1.309]	0.528	n.s.
	11∼15	55	62	0.959	[0.664, 1.386]	0.825	n.s.	8	10	0.764	[0.298, 1.959]	0.574	n.s.	19	24	0.792	[0.430, 1.457]	0.452	n.s.
	16∼	14	2	7.570	[1.718, 33.347]	0.002	∗∗	2	0	.	.	0.168	n.s.	2	5	0.400	[0.077, 2.068]	0.258	n.s.
70∼74	1∼5	4281	4271	1.130	[1.035, 1.234]	0.006	∗∗	821	822	1.029	[0.838, 1.262]	0.786	n.s.	1944	1925	1.110	[0.978, 1.261]	0.106	n.s.
	6∼10	476	356	1.508	[1.288, 1.765]	0.000	∗∗∗	88	77	1.177	[0.825, 1.679]	0.368	n.s.	209	173	1.328	[1.056, 1.671]	0.015	∗
	11∼15	55	30	2.067	[1.316, 3.247]	0.001	∗∗	5	6	0.858	[0.258, 2.851]	0.803	n.s.	26	22	1.299	[0.729, 2.317]	0.374	n.s.
	16∼	7	6	1.315	[0.441, 3.925]	0.622	n.s.	0	2	0.000	.	0.164	n.s.	4	3	1.466	[0.327, 6.577]	0.615	n.s.
65∼69	1∼5	2254	2232	1.142	[1.014, 1.286]	0.028	∗	493	487	1.167	[0.903, 1.507]	0.237	n.s.	1242	1212	1.180	[1.003, 1.389]	0.046	∗
	6∼10	224	181	1.400	[1.122, 1.747]	0.003	∗∗	52	39	1.537	[0.959, 2.463]	0.073	n.s.	114	95	1.382	[1.017, 1.879]	0.038	∗
	11∼15	42	17	2.794	[1.576, 4.955]	0.000	∗∗∗	6	6	1.153	[0.364, 3.653]	0.809	n.s.	18	15	1.382	[0.686, 2.782]	0.363	n.s.
	16∼	1	2	0.566	[0.051, 6.251]	0.637	n.s.	3	0	.	.	0.064	n.s.	2	0	.	.	0.130	n.s.
60∼64	1∼5	1081	1110	1.016	[0.844, 1.223]	0.864	n.s.	265	257	1.282	[0.903, 1.821]	0.165	n.s.	786	789	1.010	[0.815, 1.252]	0.925	n.s.
	6∼10	119	87	1.428	[1.034, 1.970]	0.030	∗	29	17	2.121	[1.083, 4.154]	0.027	∗	61	55	1.125	[0.746, 1.697]	0.575	n.s.
	11∼15	16	6	2.783	[1.073, 7.216]	0.029	∗	2	3	0.829	[0.135, 5.091]	0.839	n.s.	5	5	1.014	[0.289, 3.554]	0.982	n.s.
	16∼	0	1	0.000	.	0.328	n.s.	0	1	0.000	.	0.371	n.s.	0	0	.	.	.	·
<60	1∼5	302	277	1.440	[1.063, 1.950]	0.018	∗	97	86	1.494	[0.904, 2.471]	0.116	n.s.	261	271	0.981	[0.716, 1.343]	0.904	n.s.
	6∼10	20	10	2.641	[1.185, 5.884]	0.015	∗	7	5	1.855	[0.548, 6.276]	0.315	n.s.	23	11	2.129	[0.990, 4.578]	0.049	∗
	11∼15	1	2	0.660	[0.059, 7.381]	0.734	n.s.	0	0	.	.	.	·	0	0	.	.	.	·
	16∼	0	1	0.000	.	0.385	n.s.	0	0	.	.	.	·	0	0	.	.	.	·

Odds ratios (OR) of pulp removal for dementia within each age groups. Reference for OR was number of individuals with no pulp removed for previous 10 years (∗*P* < 0.05; ∗∗*P* < 0.01; ∗∗∗*P* < 0.001; n.s. not significant). Dmtia: Dementia, Ctrl: Control, OR: odds ratio, 95% CI: 95% confidence interval, Sig.: significance.

In contrast, the pulp removal showed limited impact on the development of vascular dementia or unspecified dementia. Removal of 6–10 pulps was associated the increase of vascular dementia in the age group of 60–64 (OR = 2.121, *P* = 0.027). No significant changes in the incidence of vascular dementia were observed in any other combination of groups. Unspecified dementia was increased by removal of 6–10 pulps in individuals under 74 (ORs between 1.3 and 2.1, *P* < 0.05), or by removal of 1–5 pulps in age group 65 to 69 (OR: 1.18, *P* = 0.046). Pulp removal did not significantly increase the ORs for unspecified dementia in individuals aged 75 and older.

### Impact of tooth extraction across age groups

Consistent with previous literatures, a high number of tooth extractions increased Alzheimer's dementia. However, the extraction of 1–5 teeth did not induce statistically significant difference in any age groups. Extraction of 6–10 teeth showed ORs ranging from 1.163 to 2.007 for individuals under 79 years of age. Extraction of 11–15 teeth resulted in ORs between 1.185 and 3.566 across all age groups, with the exception of individuals over 85, where the extraction of any number of teeth produced statistically insignificant ORs (Fig. 2 and Table 3).

**Table 3:** Odds ratios (OR) of tooth extraction for dementia.

	Tooth extracted	Alzheimer's						Vascular						Unspecified					
		Dmtia	Ctrl	OR	95% CI	*P*	Sig.	Dmtia	Ctrl	OR	95% CI	*P*	Sig.	Dmtia	Ctrl	OR	95% CI	*P*	Sig.
85 ≤	1∼5	3343	3399	0.964	[0.876, 1.061]	0.457	n.s.	454	436	1.117	[0.855, 1.460]	0.416	n.s.	988	1010	1.027	[0.864, 1.220]	0.761	n.s.
	6∼10	1249	1199	1.021	[0.911, 1.146]	0.719	n.s.	150	150	1.073	[0.776, 1.485]	0.671	n.s.	365	346	1.108	[0.899, 1.365]	0.338	n.s.
	11∼15	364	349	1.023	[0.864, 1.210]	0.795	n.s.	52	53	1.053	[0.673, 1.647]	0.822	n.s.	105	93	1.185	[0.864, 1.626]	0.291	n.s.
	16∼	123	103	1.171	[0.890, 1.541]	0.260	n.s.	12	19	0.678	[0.317, 1.448]	0.313	n.s.	40	31	1.355	[0.828, 2.216]	0.225	n.s.
80∼84	1∼5	6564	6575	1.066	[0.992, 1.145]	0.083	n.s.	1001	1026	0.942	[0.787, 1.127]	0.512	n.s.	2047	2156	0.990	[0.871, 1.125]	0.877	n.s.
	6∼10	2288	2294	1.065	[0.977, 1.160]	0.152	n.s.	325	354	0.886	[0.713, 1.102]	0.276	n.s.	742	645	1.199	[1.027, 1.400]	0.021	∗
	11∼15	714	643	1.185	[1.047, 1.342]	0.007	∗∗	125	89	1.356	[0.990, 1.856]	0.057	n.s.	210	198	1.106	[0.883, 1.384]	0.380	n.s.
	16∼	244	207	1.258	[1.035, 1.531]	0.021	∗	37	30	1.190	[0.717, 1.976]	0.500	n.s.	79	55	1.498	[1.042, 2.151]	0.028	∗
75∼79	1∼5	6834	7087	1.052	[0.980, 1.129]	0.160	n.s.	1151	1169	1.116	[0.940, 1.326]	0.210	n.s.	2475	2592	0.915	[0.813, 1.030]	0.141	n.s.
	6∼10	2205	2069	1.163	[1.066, 1.268]	0.001	∗∗	363	339	1.214	[0.982, 1.501]	0.073	n.s.	807	748	1.034	[0.895, 1.194]	0.648	n.s.
	11∼15	730	562	1.417	[1.249, 1.608]	0.000	∗∗∗	121	98	1.400	[1.030, 1.902]	0.031	∗	244	216	1.083	[0.877, 1.337]	0.459	n.s.
	16∼	251	148	1.850	[1.496, 2.289]	0.000	∗∗∗	49	36	1.543	[0.978, 2.435]	0.061	n.s.	68	68	0.959	[0.674, 1.363]	0.813	n.s.
70∼74	1∼5	4408	4635	1.020	[0.936, 1.112]	0.649	n.s.	843	925	0.855	[0.697, 1.048]	0.131	n.s.	2047	2005	1.168	[1.026, 1.330]	0.019	∗
	6∼10	1265	1133	1.198	[1.073, 1.337]	0.001	∗∗	233	199	1.098	[0.845, 1.427]	0.483	n.s.	517	518	1.142	[0.967, 1.350]	0.118	n.s.
	11∼15	376	252	1.601	[1.341, 1.910]	0.000	∗∗∗	79	54	1.372	[0.929, 2.028]	0.112	n.s.	160	127	1.442	[1.112, 1.869]	0.006	∗∗
	16∼	143	78	1.967	[1.477, 2.618]	0.000	∗∗∗	28	20	1.313	[0.719, 2.397]	0.374	n.s.	50	44	1.300	[0.854, 1.981]	0.220	n.s.
65∼69	1∼5	2311	2471	0.993	[0.883, 1.115]	0.900	n.s.	509	550	0.920	[0.717, 1.179]	0.510	n.s.	1307	1355	0.943	[0.806, 1.104]	0.468	n.s.
	6∼10	550	431	1.354	[1.152, 1.593]	0.000	∗∗∗	121	102	1.179	[0.838, 1.659]	0.345	n.s.	251	253	0.970	[0.777, 1.212]	0.791	n.s.
	11∼15	185	132	1.487	[1.164, 1.901]	0.001	∗∗	43	30	1.425	[0.852, 2.383]	0.176	n.s.	82	56	1.432	[0.993, 2.066]	0.054	n.s.
	16∼	83	51	1.727	[1.201, 2.484]	0.003	∗∗	18	10	1.789	[0.801, 3.993]	0.151	n.s.	41	26	1.542	[0.926, 2.569]	0.094	n.s.
60∼64	1∼5	1198	1261	1.054	[0.885, 1.255]	0.554	n.s.	295	315	0.830	[0.589, 1.171]	0.288	n.s.	851	878	0.956	[0.774, 1.180]	0.675	n.s.
	6∼10	269	223	1.339	[1.057, 1.695]	0.015	∗	74	65	1.009	[0.642, 1.585]	0.969	n.s.	156	154	0.999	[0.746, 1.337]	0.995	n.s.
	11∼15	81	52	1.728	[1.180, 2.531]	0.005	∗∗	17	17	0.886	[0.424, 1.854]	0.749	n.s.	41	30	1.348	[0.812, 2.238]	0.248	n.s.
	16∼	33	12	3.051	[1.548, 6.016]	0.001	∗∗	6	5	1.064	[0.312, 3.622]	0.921	n.s.	16	5	3.156	[1.136, 8.767]	0.021	∗
<60	1∼5	316	347	0.955	[0.725, 1.258]	0.744	n.s.	123	127	1.116	[0.700, 1.779]	0.645	n.s.	287	287	1.148	[0.861, 1.532]	0.346	n.s.
	6∼10	44	23	2.007	[1.153, 3.492]	0.013	∗	19	11	1.990	[0.858, 4.615]	0.105	n.s.	44	30	1.684	[1.000, 2.836]	0.049	∗
	11∼15	17	5	3.566	[1.282, 9.921]	0.010	∗	5	4	1.440	[0.365, 5.684]	0.601	n.s.	9	8	1.292	[0.484, 3.447]	0.608	n.s.
	16∼	9	4	2.360	[0.711, 7.835]	0.150	n.s.	4	2	2.304	[0.403, 13.164]	0.336	n.s.	6	2	3.445	[0.683, 17.370]	0.112	n.s.

Odds ratios (OR) of tooth extraction for dementia within each age groups. Reference for OR was number of individuals with no tooth extracted for previous 10 years (∗*P* < 0.05; ∗∗*P* < 0.01; ∗∗∗*P* < 0.001; n.s. not significant). Dmtia: Dementia, Ctrl: Control, OR: odds ratio, 95% CI: 95% confidence interval, Sig.: significance.

Similarly to pulp removal, tooth extraction did not produce significant differences in other types of dementia. The extraction of 11–15 teeth increased vascular dementia in the age group 75 to 79 (OR = 1.400, *P* < 0.05), which was the only group with significant changes. Although similar overall, tooth extraction had a greater impact on the number of unspecified dementia patients.

## Discussion

A comparative analysis of dental treatment records and dementia diagnosis records registered in the NHISS data revealed that both pulp removal and tooth extraction are associated with an increased incidence of Alzheimer's dementia. This association was not evident in patients with other types of dementia, suggesting that pulp removal or tooth extraction might specifically contribute to the development of Alzheimer's dementia rather than causing all types of dementia. The findings of the current study indicate that deterioration of pulpal tissue and tooth extraction may contribute to the development of Alzheimer's dementia, but not vascular dementia or unspecified dementia (Fig. 2).

The overall risk factors for dementia include age, genetics, general health condition, and lifestyle, which coincide with risk factors for severe dental caries or periodontal disease. Therefore, underlying health complications associated with oral conditions might have contributed to an elevated risk of cognitive decline. However, the removal of the pulp or extraction of the tooth did not increase the incidence of vascular or unspecified dementia in the majority of experimental groups. This finding strongly suggests that specific biological processes, rather than coincidental underlying conditions, might have contributed to the increased risk of Alzheimer's dementia.

It would be interesting to find the mechanism underlying contribution of pulp loss to the development of Alzheimer's dementia. The similar outcomes following pulpectomy and tooth extraction suggest that both might share a similar mechanism. Both pulpitis and periodontal disease are inflammatory disorders that often develop into chronic conditions when neglected. Proinflammatory mediators, as well as direct bacterial infections, are known to induce neuroinflammation in chronic periodontal disease, which may potentially lead to the development of Alzheimer's disease.^24^ Similarly, chronic pulpitis produces proinflammatory mediators and causes neuroinflammation, as confirmed by prolonged increases in c-Fos expression in neurons within the central nervous system.^25^ This neuroinflammatory response may be a contributing factor to the increased prevalence of Alzheimer's dementia observed in patients who have undergone larger number of pulps removed.

Given that the trigeminal nerves innervating the teeth are transected by both pulpectomy and tooth extraction, it can be postulated that nerve damage may be a significant factor shared in both processes. Dental injuries, including tooth extraction and pulpal exposure, have been demonstrated to induce c-fos expression in the central sensory pathways.^26^, ^27^, ^28^ C-fos is a proto-oncogene whose expression can be utilized as a marker of neuronal activity. It is noteworthy that elevated c-fos signaling has been documented in the hippocampal region of Alzheimer's disease patients.^29^ Consequently, the findings of the current study corroborate the hypothesis that noxious sensory experiences resulting from nerve transection during tooth extraction or pulpectomy might contribute to the pathogenesis of Alzheimer's dementia. This is consistent with previous studies suggesting that tooth loss alone is sufficient to contribute to the development of dementia.^17^

The increase in Alzheimer's dementia due to pulp removal and tooth extraction is more pronounced in younger age groups. One hypothesis is that the mechanisms underlying Alzheimer's dementia onset at a younger age may differ from those at an older age. Additionally, the increasing calcification of the pulp with age could be a contributing factor to this difference. Investigating these disparities could provide significant insights into the mechanisms of Alzheimer's disease development. Moreover, it would be beneficial to investigate whether the mechanisms linking dental issues to Alzheimer's dementia vary by age through additional research encompassing the entire cohort, rather than solely focusing on the elderly cohort.

In summary, the results indicate that pulp loss may have an impact on the development of dementia comparable to that of tooth loss. This effect was particularly pronounced in Alzheimer's dementia and was more significant with an increased number of endodontic treatments and in pre-geriatric age. Although the mechanism underlying such association could not be determined from this study and remained for further investigation, the current findings underscore the importance of the oral health and lifestyle habits that can prevent pulp loss, suggesting that early intervention of dental caries and the prevention of pulpal deterioration may help mitigate the risk of Alzheimer's dementia.

## Declaration of competing interest

The authors have no conflicts of interest relevant to this article.
